# The Impact of Plant Additives on the Quality and Safety of Ostrich Meat Sausages

**DOI:** 10.3390/molecules29133171

**Published:** 2024-07-03

**Authors:** Łukasz Woźniak, Izabela Porębska, Olga Świder, Barbara Sokołowska, Justyna Szczepańska-Stolarczyk, Krzysztof Lendzion, Krystian Marszałek

**Affiliations:** 1 Department of Food Safety and Chemical Analysis, Institute of Agricultural and Food Biotechnology—State Research Institute, 36 Rakowiecka Street, 02532 Warsaw, Poland; olga.swider@ibprs.pl; 2 Department of Microbiology, Institute of Agricultural and Food Biotechnology—State Research Institute, 36 Rakowiecka Street, 02532 Warsaw, Poland; izabela.porebska@ibprs.pl (I.P.); barbara.sokolowska@ibprs.pl (B.S.); 3 Department of Fruit and Vegetable Product Technology, Institute of Agricultural and Food Biotechnology—State Research Institute, 36 Rakowiecka Street, 02532 Warsaw, Poland; justyna.szczepanska@ibprs.pl; 4 Strusia Kraina & Mobax Sp. j., 21 Magazynowa Street, 40424 Katowice, Poland; klendzion13@gmail.com

**Keywords:** biogenic amines, food safety, meat, ostrich, plant extracts, sausage

## Abstract

Ostrich meat is an interesting alternative to poultry or beef due to its nutritional value. The addition of three plant species (hot peppers, acerola, *Schisandra chinesis*) was suggested as a method to improve the quality, safety, and consumer acceptance of sausages prepared from ostrich meat. A series of microbiological and chemical analyses (including, inter alia, content of biogenic amines, heavy metals, and bioactive compounds) of the products as well as their sensory evaluation was performed to verify this claim. The microflora of all sausages was dominated by lactic acid bacteria. The biggest threat to consumers’ health could be connected to the presence of biogenic amines formed through the enzymatic activity of lactic acid bacteria. The sausages with plant additives had better antioxidative and anti-inflammatory properties and lower fat oxidation—these features were correlated with the presence of vitamin C. Sausages with plant additives had a higher acceptability in terms of taste and smell.

## 1. Introduction

In recent years, Western societies have witnessed a significant shift in dietary patterns, driven by an increased awareness of the profound impact of nutrition on overall health. Consumers have started to look for new alternatives to traditional foods, emphasizing the importance of the nutrient content of foods as a factor in preventing chronic diseases. In the case of meat, it is usually connected with choosing meat sources characterized by lower fat content [1,2,3].

Due to its nutritional value, ostrich meat can be of interest to those who seek a healthy lifestyle. It is characterized by its high polyunsaturated fatty acid content, low saturated fatty acid [4,5], and low sodium content (32–36 mg/100 g) compared to beef (63 mg/100 g) or chicken (77 mg/100 g). It also contains the highest iron content of any other meat source available to humans (from 23.2 to 40.2 mg/kg), while beef has only about 19.3 and chicken 4–7 mg/kg [6]. Ostrich meat can be an important source of iron for patients with anemia as well as for pregnant women, and its consumption can also be beneficial for people who need to follow a low-sodium diet, such as people with hypertension. Commercially, most European ostrich meat farms have their own abattoirs, and their products are usually vacuum-packed as this provides the best microbiological protection and reduces changes in the fatty acid profile during storage [4,7].

The exploitation of herbs for the improvement of taste and microbial stability is one of the most traditional methods of meat processing [8]. Currently, in the reality of industrial-scale food production, plant material is added in the form of standardized extracts, yet the goals of the technologists remain unchanged. Among phytochemicals, phenolic compounds are often connected with beneficial health effects due to their antioxidative potential, even though the molecular mechanisms lying on a foundation of their activity remains unknown [9]. The literature includes a number of excellent review papers comprehensively describing the use of plant extracts and the quality of meat and its products [10,11,12]. Among the huge variety of plants that could be used as additives, the authors decided to choose three, based on their distinct sensory qualities and groups of bioactive compounds they contain. Acerola is predominantly sour, and it is one of the richest sources of vitamin C [13]; hot peppers are spicy and contain capsaicinoids and carotenoids [14,15]; while *Schisandra chinesis* is known as a five-flavor fruit and is a rich source of phenolic compounds [16]. The plant additives were used on two levels—one that can be considered typical for meat products and one that is higher—to evaluate the impact of a single ingredient and check if doses higher-than-typical can lead to better products. The sausages containing only meat and fat were selected as blank samples to omit the impact of other additives on the parameters of the final product.

The goal of this work was to evaluate the potential of the plant-based additives to improve the quality and safety of sausages based on ostrich meat. The addition of plant materials is often claimed by producers to improve the quality and safety of their products. Fermented sausages were selected as a matrix as they are usually considered a premium product, which justifies the use of a more expensive raw material. The scope of the work included an evaluation of product safety (both microbiological and chemical); establishing the content of distinct groups of phytochemicals; and estimating their health-promoting activities as well as consumer acceptance of the product. The authors believe that, due to the utilization of a relatively underexploited meat source and the broad scope of this work, their findings will expand the existing body of knowledge in the field of meat technology.

## 2. Results and Discussion

### 2.1. Microbiological Analyses

The microbial quality of a product is one of the most important safety factors of food products. In the case of ostrich-based sausages, the total microbial count (TMC) and the number of bacteria belonging to four groups (LAB, *Enterobacteriaceae*, *Pseudomonas*, and *Brochotrix*) were selected as criteria of analysis. The bacteria growth occurred mainly during the preparation of sausages, which lasted for circa 48 h; the counts were stable during three-week storage. The data along with statistical analysis are presented in Table 1.

Regardless of the sausage composition, lactic acid bacteria were able to dominate other bacteria groups. The counts of both TMC and LAB in all the samples were on similar level of 7.5–8.0 log CFU/g, yet statistically significant differences were observed in some cases. The LAB are generally recognized as desirable in food due to their similarity to natural gut microflora, probiotic status, and the production of metabolites that can be beneficial to host health, like short-chain fatty acids or bacteriocins [17,18].

The beneficial impact of use of plant extracts was observed in the cases of *Enterobacteriaceae* spp and *Brochotrix* spp. In the former case, the observed counts were 1.4–2.6 log CFU/g lower; in the latter, they were 0.5–1.6 log CFU/g lower. The lower levels of bacteria from those taxa can be considered an improvement as their presence leads to the spoilage of meat products [19,20,21,22]. Microbes from *Pseudomonas* spp. were not found in any of the samples.

### 2.2. Biogenic Amine Formation

**Figure 1:**
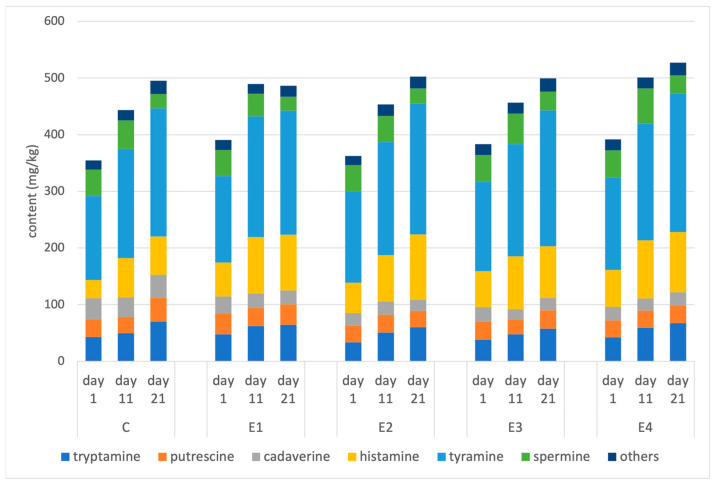
The changes in biogenic amine levels in ostrich sausages during storage. Others: sum of spermidine, agmatine, and 2-phenylethylamine. The composition of sausages is presented in Table 1.

The addition of plant extracts did not influence the content of biogenic amines in the sausages. On average, at the beginning of storage, the sausages contained 377 mg/kg of amines, which increased to 469 and 502 mg/kg after 11 and 21 days of storage, respectively. The observed increases are a result of enzyme activity: the liberation of free amino acids by endogenous proteases, their subsequent decarboxylation to amines by and in some cases, subsequent transformations by bacterial enzymes [23,24]. Tyramine was the most abundant of the amines, comprising about 40% of total content, with final levels exceeding 200 mg/kg, followed by histamine and tryptamine. An overview of the levels of individual analytes is presented in Figure 1, and the detailed data along with statistical analysis are presented in Table S1 in the Supplementary Materials.

The patterns of biogenic amines were very similar in all the samples, which can be related to lactic acid bacteria dominating the microbiota of the sausages. The chemical interactions of plant extracts with amines and their impact on the metabolism of bacteria appear to be negligible.

Despite our knowledge of biogenic amine toxicology, only levels of histamine in fish and seafood are regulated within European Union (EU) and the United States [25]. A Scientific Opinion published by the European Food Safety Authority (EFSA) did not set No-Observed-Adverse-Effects Level (NOAEL) values due to the insufficient state of knowledge, although it claimed that 50 mg of histamine and 600 mg of tyramine were not causing adverse effects in healthy individuals [26].

### 2.3. Elemental Analysis

The data on levels of analyzed metals in sausage samples are listed in Table 2.

The observed levels of iron proved the high content of this element in ostrich meat. The Daily Reference Intake for iron has been established at 14 mg/day; therefore, the consumption of one portion of product (100 g) provides over 20% of the daily demand.

The maximal levels of contaminants in food sold in the EU are set by the Commission Regulation (EU) 2023/915 [27]. The legal limits of heavy metals in meat are set at the levels of 0.100 and 0.050 mg/kg for lead and cadmium, respectively. The use of plant extracts increased levels of both lead and cadmium, although the observed contents were below legal limits for the products and therefore should not be considered a risk to consumers. Similar levels in ostrich meat from a Polish farm were observed by Rajkowska and colleagues [28]. The study of Raeeszadeh et al. conducted in an area of high environmental levels of both metals reported a median content of 0.10 and 0.18 mg/kg for lead and cadmium, respectively. The levels, however, were over 10-fold lower than in lamb and beef of the same origin [29].

### 2.4. Thiobarbituric Acid Reactive Substances (TBARS)

The oxidation of lipids occurs in meat products mainly as a result of its contact with air, increased temperatures, and enzyme activity. Oxidated lipids are not considered a threat to health of consumers, although their presence leads to a decrease in the sensory quality of the product.

The levels of TBARS in sausages have not increased significantly over time, which can be attributed to the use of vacuum packaging and maintaining the low temperature of samples. The final contents of TBARS were (all in mg of malondialdehyde equivalents per kg) 1.27 ± 0.09 for the control sausage, 0.72 ± 0.03 for sausage E2 (highest addition of acerola), and a range of 0.94–0.99 for the remaining products. Detailed results are presented in Table S2 in the Supplementary Materials.

The literature reports provide similar results. Leygonie et al. reported that vacuum storage highly inhibited TBARS production in ostrich meat [30], while Seydim and co-workers found that the addition of rosemary extract can significantly reduce TBARS levels in ostrich patties compared to a control product [31].

### 2.5. Content of Bioactive Compounds

The levels of capsaicin and dihydrocapsaicin in dried peppers were 2976 and 1142 mg/kg, respectively, which is 66,000 Scoville heat units (SHU). The sausage with the highest content of peppers (E4) had the total of ca. 60 mg/kg of capsaicinoids, while sausages with lower pepper content had approx. 8 mg/kg; the content was stable through storage. The observed contents of capsaicinoids in sausages were similar to those calculated, considering the levels in peppers and their share in sausages, proving the stability of capsaicinoids in such matrices.

Anthocyanins are naturally occurring in both acerola and *Schisandra chinesis*, although the pigments in plant extracts used for sausage production were on levels over 10-fold lower than those reported in the literature data (7.1 and 42.5 mg/L, respectively) [16,32]. The meat products did not contain measurable levels of anthocyanins. According to the literature data, the pH of raw ostrich meat is not acidic enough to ensure the stability of these compounds [33].

**Figure 2:**
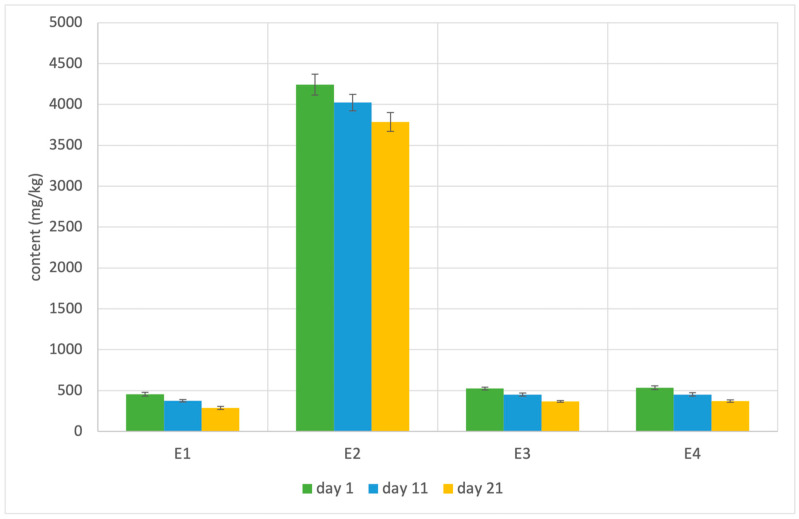
The changes in vitamin C content in ostrich sausages during storage. The composition of sausages is presented in Table 1.

Acerola extract was the only ingredient of sausages containing measurable levels of vitamin C. The observed levels were very high (134 ± 2 g/kg), as acerola is considered one of the richest sources of this vitamin in nature [13]. The content of vitamin C in sausages is shown in Figure 2. Despite the unfavorable pH of raw ostrich meat, the majority of vitamin C (69–94%) was able to survive the production process. The decrease during three weeks of storage was in the range of 11–37%, although the final concentrations were high enough for using the “high content of vitamin C” claim approved by the European Commission.

Acerola and pepper were the sources of carotenoids in the sausages [14,34,35]; the total content of these compounds in raw materials were 16.1 and 194 mg/kg, respectively. The total content of carotenoids in sausage E4 decreased during storage from 3.12 to 2.14 mg/kg, while in the rest of the sausages, levels were below the limit of quantification (0.5 mg/kg).

The data acquired during the quantification of capsaicinoids, vitamin C, and carotenoids, along with its statistical analysis, are presented in Tables S3–S5 in the Supplementary Materials.

### 2.6. Antioxidative and Anti-Inflammatory Properties

The results of assays performed to evaluate the antioxidative and anti-inflammatory potential of the meat products are presented in Table 3.

The plant additives had a significant impact on the bioactive properties of sausages. The results generally can be correlated with the content of phenolic compounds and vitamin C in sausages. The best results were obtained for sample E2, characterized by the highest level of vitamin C. In every case, we see a slight decrease in the bioactive properties of sausages during storage, which can be attributed to the degradation of phenolics and vitamin C.

### 2.7. Color

Table 4 presents an overview of the changes in the color parameters of sausages, measured with a colorimeter.

The sausages with plant components had significantly different color than the control ones. Lower values of L* as well as higher values of a* and b* were observed, which can be translated into the darker and more saturated color of the product.

In all cases, the color of sausages changed during storage, mainly due to the increase in the L* value, which can be interpreted as getting brighter. The b* parameter increased during storage, which can be interpreted as the products becoming more yellow, possibly due to fat oxidation. The a* values did not change significantly. The difference in the color that can be noticed by the consumer is usually ΔE = 1.5, which was observed in all cases.

### 2.8. Sensory Quality

The sausages were tested by panel of ten experienced sensory evaluators. The descriptor method and a ten-point scale were used. The results are listed in Table 5.

The impact of plant additives on the color of sausages that was observed with instrumental methods was confirmed by a sensory panel. The color of the control sample was rated as significantly brighter and less saturated than others, which led to a lower rate of acceptability of color. Despite the use of different amounts of plant additives, no significant changes in color were observed in samples E1–E4.

The significant differences in the smell of samples were observed for the ‘spice’ descriptor. The differences can be connected to amount of *Schisandra chinesis* fruits, characterized by a strong aroma. The panelists rated products with plant additives higher than the control sausage in the case of smell acceptability.

Taste is usually considered as the most important parameter in sensory analysis. The use of plant additives had not affected the ‘sweet’, ‘salty’, and ‘umami’ rates. The ‘sour’ score was correlated with the content of acerola extract, the ‘pungent’ score was correlated with peppers, while ‘bitter’ was correlated with *Schisandra chinesis* fruits. Generally, the acceptability of sausages with plant extracts was rated significantly higher than controls; however, in the case of E4, the pungency was too strong for most panelists, which led to a decrease in its acceptability.

The changes in the composition of samples did not significantly affect the texture of the product.

## 3. Materials and Methods

### 3.1. Research Material

Meat and fat were obtained from the meat processing company Strusia Kraina & MOBAX Sp.j. (Katowice, Poland) from 20 ostriches with a preslaughter weight of 90 to 130 kg. After overnight chilling at a temperature of −2 °C to 0 °C, approximately 30 kg ostrich meat class II (meat separated from bones, the whole carcass, without membranes and ligaments) and 3 kg ostrich fat (pure fat without membranes) were collected in three separate trials. Ingredients such as dried fruits of *Schisandra chinesis* (Nanga, Złotow, Poland), acerola (*Malpighia glabra* L.) extract standardized to 17% vitamin C (Nanga), dried hot peppers (*Capsicum annuum* L.) (Chili-Trade KFT, Batya, Hungary), natural sausage pork casings (EMAS, Dąbrowa Górnicza, Poland) and curing salt (EMAS) were purchased from a local market.

### 3.2. Sausage Formulation, Processing, and Packaging

Ostrich meat (class II) and ostrich fat were cured using a 2% (*w*/*w*) addition of curing salt. For each group, 5000 g of mixture was prepared from 4630 g (92.6%) of cured meat and 370 g (7.4%) of cured fat. The experimental groups (E1, E2, E3, and E4) contained plant additives on various levels, while the control (C) contained only meat and fat. The precise composition of the products is presented in Table 6.

**Figure 3:**
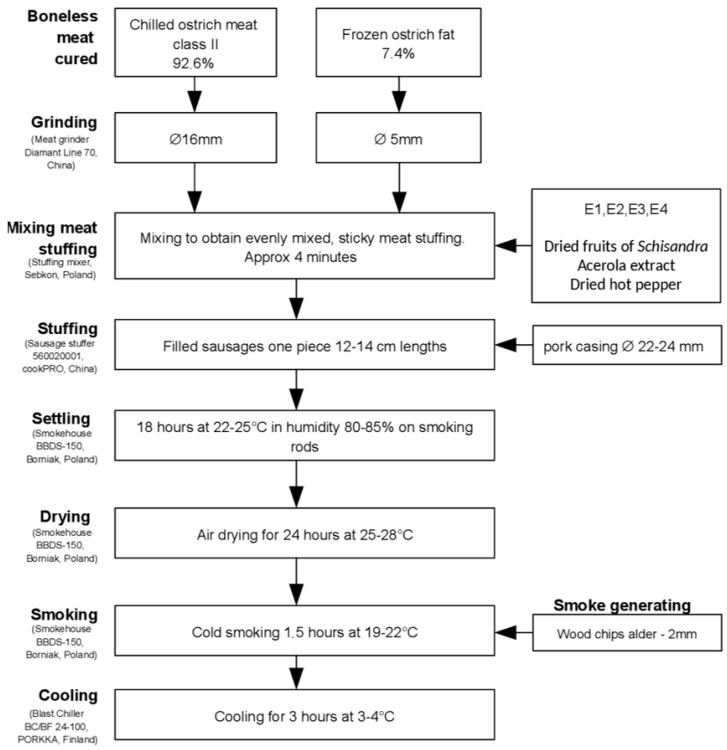
Production process of ostrich meat sausages.

The production of the experimental sausages was carried out in the research hall of the meat processing company Strusia Kraina & MOBAX Sp.j. The production process for all the groups is illustrated in Figure 3. All samples were inoculated with Primal SK soft starter culture (Van Hees, Walluf, Germany) containing *Staphylococcus carneus*.

The meat and fat used during the experiments were collected during one day of slaughtering; however, for each repetition, a separate mixture was produced to the avoid generation of pseudo-replicates.

After processing, the sausages were vacuum-packed in 40 µm multilayer barrier bags with a PET/PA/EVOH/PE structure (OTR: O_2_ 23 cc/m^2^/24 h; Bag supplier: Mercur Group, Bydgoszcz, Poland) on a vacuum machine (Tepro PP12; Tepro S.A., Koszalin, Poland), placed into sterile containers, and transported in a compressor refrigerator (CFX3 75 Dometic, Poleczki, Warsaw) to the laboratory. Then, samples were stored in the refrigerator for three weeks at a temperature of 4–5 °C. The analyses were performed on day 1, 11, and 21 of storage. All the measurements were conducted in triplicate for each group and for each length of storage.

### 3.3. Microbiological Analyses

Samples (20 g) were blended (Stomacher 400, Seward, Worthing, UK) for 2 min in 180 mL of Buffered Peptone Water (Biokar Diagnostics, Allone, France). Decimal dilutions were carried out using the same diluent. The analysis of the total microbial count (TMC), lactic acid bacteria (LABs), *Enterobacteriaceae*, *Pseudomonas*, and *Brochotrix* spp. were carried out according to ISO 4833-1:2013/Amd1:2022, ISO 15214:1998, ISO 21528-2:2017, ISO 13720:2010, and ISO 13722:2017 standards, respectively [36,37,38,39,40]. Briefly, plate count agar (PCA, Merck, Darmstadt, Germany) was used to determine total aerobic counts, MRSa (Merck, Darmstadt, Germany) was used to determine LABs, Violet Red Bile Glucose Agar (VRBG Graso Biotech, Starogard Gdański, Poland) was used to determine *Enterobacteriaceae*, Pseudomonas CFC/CN agar (Merck, Darmstadt, Germany) was used to determine *Pseudomonas,* and STAA Oxoid (Thermo Fischer, Waltham, MA, USA) was used to determine *Brochotrix*. The results were expressed as log CFU/g. Assays were performed using two independent samples. Duplicate plates were incubated under aerobic conditions. The confirmation of *Enterobacteriaceae*, *Pseudomonas*, *Brochotrix* spp and LABs was carried out according to above mentioned ISO standards (glucose fermentation test, oxidase reactions, and catalase).

### 3.4. Chemical Analyses

#### 3.4.1. Equipment and Chemicals

The content of biogenic amines and carotenoids was evaluated using an Acquity HClass ultra-high-performance liquid chromatograph coupled to an LCQ Premiere XE time-of-flight high-resolution mass spectrometer (Waters, Milford, MA, USA). Other chromatographic analyses (capsaicinoids, vitamin C, and anthocyanins) were conducted on an HPLC set consisting of a 2695 Separation Module and a 2996 PDA Detector (both Waters). The spectrophotometric analyses (TBARS, total phenolic content, DPPH scavenging, and hyaluronidase inhibition) were conducted using a Jenway 6705 UV-vis spectrophotometer (Cole-Parmer, Vernon Hills, IL, USA). The ICP-MS analyses was performed using an iCAP RQ single quadrupole spectrometer with a Kinetic Energy Discrimination chamber (Thermo Scientific, Waltham, MA, USA), while the samples were prepared with an Ethos Up microwave digestion system (Milestone, Sorisole, Italy). The solvents of a grade suitable for chromatographic analyses were supplied by Sigma-Aldrich (Saint Louis, MO, USA). The reagents were of analytical purity and were bought from various suppliers.

#### 3.4.2. Sample Preparation

The sausage samples were homogenized with a Grindomix GM 200 knife mill (Retsch GmbH, Haan, Germany) and subjected to extraction immediately after. The chemical character and stability of the sample constituents enforced the application of various extraction solvents. The method-specific protocols used for biogenic amines and TBARS are described in Section 3.4.3 and Section 3.4.4, respectively. Carotenoids were extracted with a dichloromethane (DCM)-acetone mixture (1:1, *v*/*v*) with the addition of 100 mg/L of butylated hydroxytoluene; capsaicinoids were extracted with pure DCM; and for other analyses, a water-methanol mixture (1:1, *v*/*v*) acidified to a pH of 3 with hydrochloric acid was used. The rule of thumb was to use a solid/liquid ratio of 1:25, shake the samples with three portions of the solvent, combine the obtained fractions, centrifuge them at 8000× *g* for 10 min, and filter the supernatant through cotton wool.

The content of selected bioactive compounds in the plant additives was evaluated using the same extraction methods with a solid/liquid ratio of 1:100.

#### 3.4.3. Biogenic Amines

The samples were analyzed using the protocol published by Świder et al. [41]. Two grams of the sample was shaken with an internal standard (1,7-diaminoheptane) and 40 mL of 5% trichloroacetic acid and centrifuged at 10,000× *g* for 10 min. The supernatant was filtered through a filter paper. One hundred microliters of supernatant, 1 mL of distilled water, 1 mL of borax solution (5%), and 2.5 mL of 20 mM dansyl chloride solution (20 mM in acetonitrile) were mixed in a 15 mL polypropylene tube and shaken in a water bath operated at 30 °C for 1 h without light access. Next, 10 μL of formic acid was added, and the tube was left intact for 15 min in a dark place. Finally, the mixture was filtered through a 0.45 μm syringe filter into a chromatographic vial.

The analytes were separated on a Cortex UPLC C18 column, 1.6 µm, 2.1 × 100 mm (Waters), using gradient of water-acetonitrile mixtures (90:10 *v*/*v*, 10:90 *v*/*v*), each including 5 mM of ammonium formate and 1% of formic acid. The analytes were quantified using heated electrospray ionization working in positive polarization mode with external calibration curves of the standards of nine biogenic amines (range of 1–1000 µg/L). The technical details of both chromatographic separation and spectrometric analysis are presented in the original protocol [41].

#### 3.4.4. Content of Selected Metals (Iron, Cadmium, and Lead)

The portions of homogenized product of approximately 0.25 g were mixed with 5 mL of concentrated nitric acid and 0.5 mL of hydrogen peroxide (30%) in mineralization vessels. After 15 min of preliminary reaction, the vessels were placed in a digestion system, heated to 210 °C within 20 min, and kept at this temperature for another 20 min. The samples were transferred to PFA volumetric flasks, and the solution of yttrium (final concentration of 1 μg/kg) was added as an internal standard. The content of the following isotopes was quantified with ICP-MS working in the KED mode: ^57^Fe, ^111^Cd, and ^208^Pb. A standard addition method was used.

#### 3.4.5. Thiobarbituric Acid Reactive Substances (TBARS)

The content of TBARS was estimated using the method presented by Maraschiello et al. [42]. Two grams of sausage homogenate was combined with 20 mL of cold 25% trichloroacetic acid, shaken for 15 min and centrifuged at 12,000× *g* for 15 min. Two milliliters of supernatant was mixed with 1 mL of 0.6% thiobarbituric acid and incubated for 30 min at a temperature of 70 °C. After cooling, an absorbance was recorded at the wavelength of 532 nm and compared with the malonaldehyde-based calibration curve. Malondialdehyde (MDA) was used as a standard to prepare a calibration curve in the range of 0.2–10 mg/kg.

#### 3.4.6. Capsaicinoids

The content of capsaicin and related compounds was quantified using the application note of Waite and Aubin [15]. The DCM extract was evaporated to dryness with a rotary evaporator, reconstituted in methanol, and passed through a 0.45 μm syringe filter. A gradient of methanol and water was used for the separation of 25 μL samples on a Sunfire C18 5 μm, 4.6 × 250 mm column (Waters) kept at a temperature of 40 °C. The absorbance at a wavelength of 281 nm was used for quantitative purposes along with calibration curves of capsaicin and didhdrocapsaicin (range of 0.01–10 mg/L).

#### 3.4.7. Vitamin C

The content of two vitamin C forms, L-ascorbic (AA) and L-dehydroascorbic acid (DHAA), was measured according to the method proposed by Odriozola-Serrano et al. [43]. For the analysis, 1 mL of the sample was diluted with 1 mL of 0.01% phosphoric acid (determination of AA) or 1 mL of 1 g/L solution of dithiothreitol (DTT) in 0.01% phosphoric acid (determination of AA and DHAA sum). The samples with DTT were kept in a temperature of 4 °C for at least 1 h before the injection. Separation was performed on a Sunfire C18, 5 μm, 4.6 mm × 250 mm analytical column (Waters). Analysis of the 10 μL samples was performed at an isocratic flow of 0.01% phosphoric acid at a rate of 1.0 mL min^−1^ and a column temperature of 25 °C. The analytes were quantified using absorption at a wavelength of 245 nm and an external calibration curve of AA (range of 0.1–100 mg/L).

#### 3.4.8. Carotenoids

The content of carotenoids was quantified using the protocol presented by Janiszewska-Turak and co-authors [35]. The extracts were evaporated to dryness with a rotary evaporator, reconstituted in isopropanol and passed through a 0.2 μm syringe filter. Samples were separated on a Cortex UPLC C18 column, 1.6 µm, 2.1 × 100 mm (Waters), which was eluted at a flow rate of 0.4 mL/min using a gradient flow. The mass spectrometer was fitted with an atmospheric pressure chemical ionization (APCI) source, and positive mass spectra were collected. The carotenoids and their esters were identified using the data from the paper of Schweiggert et al. [44]. The technical details of both chromatographic separation and spectrometric analysis are presented in the original protocol. The compounds were identified using their mass spectra and data from our earlier experiments [35].

#### 3.4.9. Anthocyanins

The content of anthocyanins was evaluated with a method described by Oszmiański [45]. A Sunfire C18, 5 μm, 4.6 mm × 250 mm analytical column (Waters) was used. The separation of the samples was performed using a gradient of 80% solution of acetonitrile in 4.5% formic acid (solvent A) and 4.5% formic acid (solvent B) within 26 min at a flow rate of 1 mL/min and a column temperature of 25 °C. The analytes were quantified using absorption at a wavelength of 520 nm; the results were expressed as equivalents of cyanidin-3-glucoside (Cy-3-Glu). Cy-3-Glu was used as an external standard to prepare a calibration curve in the range of 1–100 mg/kg.

#### 3.4.10. Total Phenolic Content (TPC)

The spectrophotometric method described by Gao et al. [46] was used for the evaluation of total phenolic content. The reaction mixtures composed of 0.1 mL of sample, 2.0 mL of water, 0.2 mL of Folin-Ciocalteu reagent, and 1.0 mL of sodium carbonate solution (15%) were incubated for 2 h at room temperature. The phenolic content was determined by measuring absorbance at a wavelength of 765 nm. The results were expressed as equivalents of gallic acid.

#### 3.4.11. DPPH Scavenging

The determination of scavenging activity against DPPH radicals was carried out with a method described by Yen and Chen [47]. The 0.1 mL samples were combined with 2 mL of 1 mM DPPH solution (1 mM) and incubated at room temperature for 30 min. The degradation of violet-pigmented radicals was evaluated spectrophotometrically at 517 nm. The results were expressed as equivalents of Trolox.

#### 3.4.12. Hyaluronidase Inhibition

The anti-inflammatory activity of the samples was evaluated by an hyaluronidase inhibition assay presented by Osés et al. [48]. The reaction media were prepared by mixing 70 μL of hyaluronic acid from *Streptococcus equi* solution (5 g/L), 100 μL of pH 3.68 buffer (0.2 M of sodium formate, 0.1 M of sodium chloride, 2 g/L of bovine serum albumin; pH adjusted with formic acid), 200 μL of water and 50 μL of the sample. The mixture was preheated to 37 °C and the reaction was initiated by the addition of 50 μL of hyaluronidase from bovine testes type IV-S (600 U/mL; dissolved in 0.9% NaCl). The mixture was incubated for 1 h at a temperature of 37 °C, and afterwards, the enzymatic reaction was stopped by the addition of 100 μL of 0.8 M potassium tetraborate and a 3 min incubation in boiling water. After cooling to room temperature, 750 μL of *p*-dimethylaminobenzaldehyde solution (3.3%) in a mixture of 10 N HCl and glacial acetic acid (1:23 *v*/*v*) was added to each sample. The samples were incubated at a temperature of 37 °C, and the absorbance was measured at a wavelength of 586 nm. The results were expressed as a percentage of inhibition.

### 3.5. Other Analyses

#### 3.5.1. Color

The determination of the color in the L*a*b* scale was performed with a ColorQuest XE (HunterLab, Reston, VA, USA) colorimeter equipped with a xenon flashlamp. Determination was performed according to the apparatus instructions in reflection mode, using a D65 light source and a 10° observer. The cross-sections of the sausages were analyzed in six replications.

#### 3.5.2. Sensory Evaluation

The sensory quality of the products was tested by the group of 10 panelists on the last day of storage. The panelists were members of a specialized group meeting the requirements of the ISO/IEC 17025 standard [49]. Twenty-three descriptors were evaluated using ten-point scale system. The descriptor list is presented in Table 7.

### 3.6. Statistical Analysis

Data were analyzed using Statistica 7.1 software (StatSoft, Tulsa, OK, USA). The statistical significance of the differences in mean values was determined using an ANOVA test at α = 0.05, and when significant differences existed, a Tukey test was performed.

## 4. Conclusions

The presented results emphasize the superiority of plant-containing sausages over the control sample in terms of quality and the possible beneficial effects on consumer health. The safety, both chemical and microbiological, of all products can be considered similar.

The main safety concern is connected with the presence of biogenic amines at levels reaching up to 500 mg/kg. The total content in all variants was at a similar level, while subtle differences in the observed patterns of amines do not affect general safety. This type of threat can be considered typical for food from the ‘fermented sausage’ category, although measures should be taken to ameliorate it, such as by using lactic acid bacteria strains with a low potential of biogenic amine formation. The plant additives also lowered levels of bacteria from *Enterobacteriacea* and *Brochotix* spp. in the product; however, the dominance of lactic acid bacteria in the matrix make microbiological safety of less concern.

The presence of various groups of bioactive compounds of products led to an increase in their antioxidative and anti-inflammatory properties. Even though only vitamin C has a well-established portfolio of health-related benefits, the presence of compounds, like phenolic compounds, is recommended by the majority of the scientific community and is well-perceived by consumers. The analysis of the obtained data may suggest that the highest potential is exhibited by vitamin C, present in acerola extract.

The use of plant additives also resulted in the higher sensory quality of the products. A darker and more saturated color of the sausages was claimed by the sensory panel, as well as a more sophisticated smell and taste of the product. In the case of E4 sausages, the content of capsaicinoids was too high, which resulted in decreased rates. The higher oxidation of fat, shown by TBARS analysis, was not detectable by the expert panel.

## Figures and Tables

**Table 1:** Microbial quality of sausages during storage. All results are in log CFU/g. The composition of sausages is presented in Section 3, Different letters denote groups that are significantly different at the 0.05 significance level according to Tukey’s HSD test (two-way ANOVA).

	Day	C	E1	E2	E3	E4
Total microbial count (TMC)	1	6.88 ± 0.12 f	7.67 ± 0.19 cd	7.77 ± 0.13 bc	7.48 ± 0.12 e	7.60 ± 0.08 d
	11	6.93 ± 0.15 f	7.60 ± 0.06 d	7.78 ± 0.12 bc	7.65 ± 0.04 cd	7.58 ± 0.05 de
	21	7.78 ± 0.08 bc	7.90 ± 0.06 b	7.73 ± 0.02 c	8.00 ± 0.01 a	8.00 ± 0.03 a
Lactic acid bacteria (LAB)	1	6.70 ± 0.09 g	7.59 ± 0.04 de	7.82 ± 0.05 bc	7.20 ± 0.12 f	7.76 ± 0.15 bc
	11	6.61 ± 0.02 g	7.59 ± 0.12 de	7.86 ± 0.07 b	7.53 ± 0.02 e	7.70 ± 0.07 cd
	21	7.62 ± 0.04 d	7.80 ± 0.08 bc	7.81 ± 0.07 bc	8.04 ± 0.09 a	7.88 ± 0.07 b
*Enterobacteriaceae* spp.	1	5.32 ± 0.18 a	3.32 ± 0.10 c	2.83 ± 0.04 e	2.58 ± 0.12	3.51 ± 0.12 c
	11	4.60 ± 0.11 b	3.01 ± 0.08 d	1.80 ± 0.12 g	2.10 ± 0.03 f	3.04 ± 0.12 d
	21	4.41 ± 0.06 b	2.85 ± 0.03 e	1.85 ± 0.06 g	2.20 ± 0.08 f	2.99 ± 0.02 d
*Pseudomonas* spp.	1	2.26 ± 0.27 a	<1.00	<1.00	<1.00	<1.00
	11	2.26 ± 0.03 a	<1.00	<1.00	<1.00	<1.00
	21	<1.00	<1.00	<1.00	<1.00	<1.00
*Brochotrix* spp.	1	4.58 ± 0.01 b	4.08 ± 0.02 d	2.96 ± 0.16	4.79 ± 0.14 a	3.93 ± 0.10 e
	11	4.23 ± 0.12 c	3.53 ± 0.12 g	2.45 ± 0.11 i	3.25 ± 0.19 h	3.95 ± 0.10 e
	21	4.18 ± 0.01 c	3.70 ± 0.10 f	2.60 ± 0.04 i	3.40 ± 0.04 gh	3.61 ± 0.05 g

**Table 2:** Content of iron, cadmium, and lead in sausages. Different letters denote groups that are significantly different at the 0.05 significance level according to Tukey’s HSD test (the comparison of variants). All results are in mg/kg. The composition of sausages is presented in Section 3.

	Fe	Cd	Pb
C	30.5 ± 1.5 a	0.002 ± 0.001 a	0.011 ± 0.001 d
E1	29.5 ± 1.5 a	0.003 ± 0.001 a	0.013 ± 0.001 c
E2	28.4 ± 1.4 a	0.003 ± 0.001 a	0.016 ± 0.001 ab
E3	30.3 ± 1.5 a	0.004 ± 0.001 a	0.018 ± 0.001 a
E4	31.0 ± 1.5 a	0.003 ± 0.001 a	0.015 ± 0.001 b

**Table 3:** Changes in antioxidative and anti-inflammatory features of ostrich sausages during storage. Abbr.: TPC = total phenolic content. Different letters denote groups that are significantly different at the 0.05 significance level according to Tukey’s HSD test (two-way ANOVA). The composition of sausages is presented in Section 3.

		TPC (μg Gallic Acid/g)	DPPH Scavenging (μmol Trolox/g)	Hyaluronidase Inhibition (%)
C	day 1	4 ± 2 i	0.9 ± 0.2 e	0.4 ± 0.2 c
	day 11	4 ± 1 i	0.6 ± 0.2 e	0.3 ± 0.3 c
	day 21	4 ± 2 i	0.7 ± 0.3 e	0.3 ± 0.2 c
E1	day 1	148 ± 8 fg	7.0 ± 0.5 d	4.2 ± 0.6 b
	day 11	133 ± 8 g	6.3 ± 0.3 d	3.6 ± 0.4 b
	day 21	116 ± 9 h	6.5 ± 0.3 d	3.4 ± 0.6 b
E2	day 1	966 ± 39 a	22.3 ± 1.5 a	11.5 ± 0.9 a
	day 11	903 ± 40 a	19.7 ± 1.0 b	10.6 ± 0.4 a
	day 21	826 ± 17 b	18.7 ± 1.0 b	10.3 ± 0.8 a
E3	day 1	289 ± 16 c	13.0 ± 0.6 c	4.4 ± 0.5 b
	day 11	243 ± 12 d	12.6 ± 1.1 c	3.9 ± 0.5 b
	day 21	224 ± 14 d	12.0 ± 0.7 c	4.1 ± 0.3 b
E4	day 1	188 ± 11 e	6.8 ± 0.8 d	4.0 ± 0.3 b
	day 11	165 ± 9 f	6.6 ± 0.4 d	3.8 ± 0.5 b
	day 21	155 ± 9 f	6.1 ± 0.3 d	3.8 ± 0.5 b

**Table 4:** Changes in color parameters of ostrich sausage cross-sections during storage. The composition of sausages is presented in Section 3. Symbols: L*, a*, b*—color parameters, ΔE—color difference (as compared to day 1).

		L*	a*	b*	ΔE
C	day 1	39.08 ± 1.62	11.35 ± 1.14	11.58 ± 1.15	-
	day 11	41.09 ± 2.22	12.34 ± 0.69	12.85 ± 1.04	2.58
	day 21	43.93 ± 1.22	12.14 ± 0.85	13.83 ± 1.42	5.40
E1	day 1	35.90 ± 0.65	16.35 ± 1.26	13.09 ± 0.95	-
	day 11	37.98 ± 1.43	15.90 ± 1.00	13.43 ± 1.65	2.16
	day 21	39.68 ± 2.04	15.80 ± 0.88	13.79 ± 1.50	3.88
E2	day 1	35.85 ± 0.85	17.55 ± 0.65	12.73 ± 0.96	-
	day 11	37.09 ± 1.27	16.75 ± 0.49	13.01 ± 1.43	1.50
	day 21	38.68 ± 1.90	16.93 ± 1.13	13.09 ± 0.98	2.92
E3	day 1	36.85 ± 0.72	15.40 ± 1.43	12.78 ± 1.45	-
	day 11	37.95 ± 1.70	14.66 ± 0.53	13.61 ± 1.15	1.56
	day 21	39.30 ± 1.52	14.81 ± 0.73	14.80 ± 1.25	3.23
E4	day 1	36.63 ± 1.15	16.03 ± 1.52	12.97 ± 1.18	-
	day 11	37.98 ± 1.35	15.93 ± 0.65	13.58 ± 1.23	1.49
	day 21	39.08 ± 1.20	15.84 ± 0.67	14.31 ± 0.93	2.80

**Table 5:** Sensory analysis of sausages after 21 days of storage. All results are on a 0–10 scale, where 10 = the highest/the most intensive and 1 = the lowest/the least intensive. Different letters denote groups that are significantly different at the 0.05 significance level according to Tukey’s HSD test (the comparison of variants). The composition of sausages is presented in Section 3.

		C	E1	E2	E3	E4
color	brightness	6.3 a	5.3 b	5.2 b	5.3 b	5.3 b
	saturation	4.2 b	5.6 a	5.7 a	5.4 a	5.6 a
	homogeneity	6.3 a	6.1 a	6.5 a	6.2 a	6.1 a
	acceptability	6.8 b	8.2 a	8.3 a	8.3 a	8.1 a
smell	meat	7.0 a	6.8 a	7.1 a	6.7 a	6.9 a
	smoked	6.1 a	6.2 a	5.8 a	5.8 a	5.9 a
	spice	1.1 c	3.2 b	3.1 b	6.8 a	3.3 b
	rancid	0.8 a	0.4 b	0.2 b	0.3 b	0.4 b
	acceptability	7.3 b	8.2 a	8.1 a	8.5 a	8.2 a
taste	sweet	1.4 a	1.5 a	1.2 a	1.3 a	1.3 a
	salty	3.2 a	3.0 a	3.3 a	3.4 a	3.4 a
	sour	2.1 c	3.3 b	5.2 a	3.5 b	3.3 b
	bitter	0.7 c	1.2 bc	1.1 bc	2.1 a	1.4 b
	umami	7.2 a	7.1 a	7.4 a	7.1 a	7.0 a
	pungent	1.4 c	3.2 b	3.1 b	3.6 b	9.2 a
	acceptability	6.4 b	7.6 a	7.3 a	7.1 ab	4.1 c
texture	homogeneity	6.4 a	6.3 a	6.4 a	6.2 a	6.2 a
	juiciness	3.9 a	4.1 a	4.1 a	3.8 a	4.2 a
	chewiness	8.1 a	7.9 a	8.2 a	8.2 a	8.2 a
	hardness	7.6 a	7.4 a	7.3 a	7.4 a	7.4 a
	acceptability	7.1 a	7.2 a	7.0 a	7.2 a	7.2 a

**Table 6:** Composition of sausage samples. Percentages in italics represent the relative addition of the plant ingredients.

Ingredients	C	E1	E2	E3	E4
cured meat and fat mixture	5000 g	5000 g	5000 g	5000 g	5000 g
*Schisandra chinesis* fruits	-	10 g 0.2%	10 g 0.2%	50 g 1.0%	10 g 0.2%
acerola extract	-	25 g 0.5%	175 g 3.5%	25 g 0.5%	25 g 0.5%
dried hot peppers	-	10 g 0.2%	10 g 0.2%	10 g 0.2%	75 g 1.5%

**Table 7:** The descriptor list for sensory evaluation of ostrich meat sausages. The features were assessed on a ten-point scale (10—the highest/the most intensive, 0—the lowest/the least intensive).

Color	Smell	Taste	Texture
brightness	meat	sweet	homogeneity
saturation	smoked	salty	juiciness
homogeneity	spice	sour	chewiness
overall acceptability	rancid	bitter	hardness
	overall acceptability	umami	overall acceptability
		pungent	
		overall acceptability	

## Data Availability

The original contributions presented in the study are included in the article and Supplementary Material, further inquiries can be directed to the corresponding authors.

## Supplementary Materials

The following are available online at https://www.mdpi.com/article/10.3390/molecules29133171/s1, Table S1: The content of biogenic amines in ostrich sausages during storage, Table S2: The content of TBARS (thiobarbituric acid reactive substances) in ostrich sausages during storage, Table S3: The total content of capsaicinoids in ostrich sausages during storage, Table S4: The content of vitamin C in ostrich sausages during storage, Table S5: The total content of carotenoids in ostrich sausages during storage.
