# Supplementary material for: The Impact of Plant Additives on the Quality and Safety of Ostrich Meat Sausages
Source: Molecules. 2024 Jul 3;29(13):3171. doi: 10.3390/molecules29133171 (PMC11243488; doi:10.3390/molecules29133171)
Supplement: Supplementary file 1 [file molecules-29-03171-s001.zip › molecules-2981583-supplementary.pdf]

Supplementary Material

Table S1. The content of biogenic amines in ostrich sausages during storage. Different letters denote groups that are significantly different at the 0.05 significance level according to Tukey's HSD test (two-way ANOVA). All results in mg/kg.

	day	C	E1	E2	E3	E4
tryptamine	1	42.9 ± 1.7 *f*	47.9 ± 1.3 *e*	33.6 ± 1.6 *h*	38.4 ± 0.5 *g*	42.3 ± 2.2 *f*
	11	49.6 ± 3.3 *e*	62.2 ± 2.8 *c*	50.8 ± 3.2 *e*	47.7 ± 3.6 *e*	59.1 ± 2.1 *cd*
	21	70.1 ± 4.2 *a*	64.4 ± 2.9 *bc*	60.3 ± 3.1 *cd*	57.4 ± 1.8 *d*	67.4 ± 3.3 *ab*
putrescine	1	31.3 ± 1.9 *cde*	36.8 ± 1.5 *b*	29.3 ± 1.9 *def*	31.7 ± 0.8 *cd*	29.8 ± 1.5 *de*
	11	28.9 ± 1.7 *def*	32.0 ± 1.2 *c*	31.3 ± 1.7 *cde*	26.3 ± 1.6 *f*	30.3 ± 1.7 *cde*
	21	41.6 ± 1.1 *a*	35.9 ± 0.8 *b*	28.6 ± 1.4 *ef*	32.5 ± 2.0 *cd*	31.6 ± 0.8 *cd*
cadaverine	1	37.1 ± 2.2 *b*	29.8 ± 1.2 *c*	22.1 ± 0.6 *fg*	25.4 ± 0.6 *d*	23.9 ± 1.4 *def*
	11	34.3 ± 1.1 *b*	25.4 ± 1.1 *d*	23.6 ± 1.0 *def*	19.3 ± 1.5 *h*	21.3 ± 1.4 *gh*
	21	41.2 ± 0.9 *a*	24.4 ± 0.7 *de*	19.9 ± 0.5 *h*	21.1 ± 1.9 *gh*	23.1 ± 0.8 *efg*
histamine	1	32.0 ± 0.3 *j*	59.9 ± 1.1 *h*	53.5 ± 2.4 *i*	63.3 ± 2.5 *g*	65.1 ± 3.2 *fg*
	11	69.1 ± 1.4 *f*	99.6 ± 4.5 *c*	81.3 ± 3.7 *e*	92.6 ± 2.2 *d*	102.6 ± 3.9 *bc*
	21	67.4 ± 3.0 *f*	98.6 ± 1.7 *c*	115.2 ± 4.0 *a*	91.2 ± 1.6 *d*	106.0 ± 2.2 *b*
tyramine	1	148.8 ± 3.9 *g*	152.4 ± 2.9 *g*	161.9 ± 6.1 *f*	158.0 ± 4.4 *fg*	163.9 ± 5.1 *f*
	11	193.1 ± 4.9 *e*	213.2 ± 9.0 *c*	200.1 ± 11.2 *de*	198.1 ± 8.8 *de*	205.9 ± 3.5 *cd*
	21	226.5 ± 4.7 *b*	219.4 ± 8.1 *bc*	230.6 ± 12.4 *ab*	240.0 ± 9.1 *a*	245.0 ± 10.9 *a*
spermine	1	46.3 ± 1.1 *d*	46.2 ± 1.4 *d*	45.5 ± 2.4 *d*	47.3 ± 0.5 *cd*	47.6 ± 2.3 *cd*
	11	50.3 ± 4.4 *bc*	39.8 ± 3.2 *e*	46.1 ± 2.0 *d*	54.3 ± 2.2 *b*	62.2 ± 3.0 *a*
	21	25.1 ± 2.4 *g*	24.3 ± 1.4 *g*	26.8 ± 1.2 *g*	32.7 ± 1.5 *f*	31.5 ± 1.0 *f*
spermidine	1	4.9 ± 0.2 *gh*	5.1 ± 0.3 *efg*	5.8 ± 0.1 *cde*	5.5 ± 0.1 *def*	5.3 ± 0.3 *efg*
	11	5.3 ± 0.2 *efg*	4.6 ± 0.7 *h*	5.5 ± 0.1 *def*	5.8 ± 0.4 *cde*	6.1 ± 0.2 *bcd*
	21	5.2 ± 0.4 *efg*	5.2 ± 0.4 *efg*	6.8 ± 0.3 *a*	7.4 ± 0.6 *a*	6.4 ± 0.4 *ab*
2-phenylethylamine	1	11.1 ± 0.8 *g*	12.2 ± 0.3 *f*	10.2 ± 0.3 *h*	13.7 ± 0.3 *d*	14.1 ± 0.7 *cd*
	11	12.4 ± 0.8 *fg*	12.7 ± 0.7 *def*	14.8 ± 1.1 *c*	13.2 ± 1.2 *de*	13.4 ± 0.7 *de*
	21	18.2 ± 0.7 *a*	13.8 ± 0.9 *cd*	13.9 ± 1.0 *cd*	16.3 ± 1.2 *b*	16.2 ± 0.9 *b*

Table S2. The content of TBARS (thiobarbituric acid reactive substances) in ostrich sausages during storage. Different letters denote groups that are significantly different at the 0.05 significance level according to Tukey's HSD test (two-way ANOVA). All results in mg of malonaldehyde equivalents per kg.

	**day 1**	**day 11**	**day 21**
C	1.22 ± 0.06 *a*	1.21 ± 0.08 *a*	1.27 ± 0.09 *a*
E1	0.96 ± 0.05 *b*	1.01 ± 0.03 *b*	0.99 ± 0.05 *b*
E2	0.70 ± 0.03 *c*	0.70 ± 0.02 *c*	0.72 ± 0.03 *c*
E3	0.92 ± 0.05 *b*	0.95 ± 0.04 *b*	0.94 ± 0.03 *b*
E4	0.96 ± 0.03 *b*	0.95 ± 0.05 *b*	0.95 ± 0.05 *b*

Table S3. The total content of capsaicinoids in ostrich sausages during storage. Different letters denote groups that are significantly different at the 0.05 significance level according to Tukey's HSD test (two-way ANOVA). All results in mg/kg.

	**day 1**	**day 11**	**day 21**
C	< 1.0	< 1.0	< 1.0
E1	7.7 ± 0.5 *b*	8.1 ± 0.5 *b*	7.7 ± 0.6 *b*
E2	7.8 ± 0.5 *b*	7.6 ± 0.7 *b*	8.1 ± 0.7 *b*
E3	8.3 ± 0.6 *b*	7.8 ± 0.4 *b*	7.8 ± 0.3 *b*
E4	60.2 ± 2.8 *a*	61.0 ± 2.5 *a*	60.2 ± 2.5 *a*

Table S4. The content of vitamin C in ostrich sausages during storage. Different letters denote groups that are significantly different at the 0.05 significance level according to Tukey's HSD test (two-way ANOVA). All results in mg/kg.

	**day 1**	**day 11**	**day 21**
C	< 10	< 10	< 10
E1	456 ± 24 *e*	375 ± 16 f	288 ± 18 *g*
E2	4243 ± 129 *a*	4024 ± 101 *b*	3784 ± 115 *c*
E3	524 ± 17 *d*	449 ± 19 *e*	367 ± 9 *f*
E4	536 ± 23 *d*	451 ± 21 *e*	370 ± 14 *f*

Table S5. The total content of carotenoids in ostrich sausages during storage. Different letters denote groups that are significantly different at the 0.05 significance level according to Tukey's HSD test (two-way ANOVA). All results in mg/kg.

	**day 1**	**day 11**	**day 21**
C	< 0.50	< 0.50	< 0.50
E1	< 0.50	< 0.50	< 0.50
E2	< 0.50	< 0.50	< 0.50
E3	< 0.50	< 0.50	< 0.50
E4	3.12 ± 0.15 *a*	2.60 ± 0.13 *b*	2.14 ± 0.22 *c*
